# Optimising participation and generalisability: the use of opt-out recruitment for an implementation trial in primary care

**DOI:** 10.1186/1745-6215-16-S2-O76

**Published:** 2015-11-16

**Authors:** Suzanne Hartley, Paul Carder, Robbie Foy, Peter Heudtlass, Liz Glidewell, Emma Ingleson, Stella Johnson, Paul Lord, Tom Willis, Amanda Farrin

**Affiliations:** 1 University of Leeds, Leeds, UK; 2 NHS Yorkshire and Humber Commissioning Support, Bradford, UK

## 

Action to Support Practices Implementing Research Evidence (ASPIRE) is an NIHR-funded programme aiming to develop and evaluate interventions to promote the uptake of NICE recommendations in general practice. Multifaceted intervention packages targeting key recommendations will be evaluated using anonymised, routinely collected electronic health records in two parallel cluster randomised controlled trials (cRCTs) in 144 general practices across West Yorkshire. We will replicate the ‘real-life’ conditions under which quality improvement initiatives are conducted: ensuring that a wide range of general practices typically targeted by such initiatives is therefore critical to the generalisability of the trial findings.

To eliminate the risk of over-estimating the quality of care from self-selected, motivated practices, we used an opt-out recruitment approach, where eligible practices were informed of the research and excluded only if they confirmed they were unwilling to participate.

Fifty-six of the 242 eligible practices opted-out and eight were excluded for other reasons. One hundred and seventy eight practices were randomised. Consequently, the programme exceeded its original target sample sizes for the trials at greater efficiency than the alternative, traditional opt-in method.

We will share our rationale for this recruitment approach and present reasons why practices chose to opt-out. We will present aggregate practice level data to compare characteristics of the recruited and non-recruited practices to demonstrate the generalisability of the findings. We propose that the use of opt-out in ‘low-risk’ research, particularly implementation research, can increase both participation and generalisability of the findings. It also provides an efficient use of resource.

